# Ultra-rare *RTEL1* gene variants associate with acute severity of COVID-19 and evolution to pulmonary fibrosis as a specific long COVID disorder

**DOI:** 10.1186/s12931-023-02458-7

**Published:** 2023-06-16

**Authors:** Laura Bergantini, Margherita Baldassarri, Miriana d’Alessandro, Giulia Brunelli, Gaia Fabbri, Kristina Zguro, Andrea Degl’Innocenti, GEN-COVID Multicenter study, Francesca Mari, Sergio Daga, Ilaria Meloni, Mirella Bruttini, Susanna Croci, Mirjam Lista, Debora Maffeo, Elena Pasquinelli, Viola Bianca Serio, Enrica Antolini, Simona Letizia Basso, Samantha Minetto, Rossella Tita, Maria Antonietta Mencarelli, Caterina Lo Rizzo, Anna Maria Pinto, Francesca Ariani, Francesca Montagnani, Mario Tumbarello, Ilaria Rancan, Massimiliano Fabbiani, Paolo Cameli, David Bennett, Federico Anedda, Simona Marcantonio, Sabino Scolletta, Federico Franchi, Maria Antonietta Mazzei, Susanna Guerrini, Edoardo Conticini, Luca Cantarini, Bruno Frediani, Danilo Tacconi, Chiara Spertilli Raffaelli, Arianna Emiliozzi, Marco Feri, Alice Donati, Raffaele Scala, Luca Guidelli, Genni Spargi, Marta Corridi, Cesira Nencioni, Leonardo Croci, Gian Piero Caldarelli, Davide Romani, Paolo Piacentini, Maria Bandini, Elena Desanctis, Silvia Cappelli, Anna Canaccini, Agnese Verzuri, Valentina Anemoli, Manola Pisani, Agostino Ognibene, Maria Lorubbio, Alessandro Pancrazzi, Massimo Vaghi, Antonella D.’Arminio Monforte, Federica Gaia Miraglia, Mario U. Mondelli, Stefania Mantovani, Raffaele Bruno, Marco Vecchia, Marcello Maffezzoni, Enrico Martinelli, Massimo Girardis, Stefano Busani, Sophie Venturelli, Andrea Cossarizza, Andrea Antinori, Alessandra Vergori, Stefano Rusconi, Matteo Siano, Arianna Gabrieli, Agostino Riva, Daniela Francisci, Elisabetta Schiaroli, Carlo Pallotto, Saverio Giuseppe Parisi, Monica Basso, Sandro Panese, Stefano Baratti, Pier Giorgio Scotton, Francesca Andretta, Mario Giobbia, Renzo Scaggiante, Francesca Gatti, Francesco Castelli, Eugenia Quiros-Roldan, Melania Degli Antoni, Isabella Zanella, Matteo della Monica, Carmelo Piscopo, Mario Capasso, Roberta Russo, Immacolata Andolfo, Achille Iolascon, Giuseppe Fiorentino, Massimo Carella, Marco Castori, Giuseppe Merla, Gabriella Maria Squeo, Filippo Aucella, Pamela Raggi, Rita Perna, Matteo Bassetti, Antonio Di Biagio, Maurizio Sanguinetti, Luca Masucci, Alessandra Guarnaccia, Serafina Valente, Alex Di Florio, Marco Mandalà, Alessia Giorli, Lorenzo Salerni, Patrizia Zucchi, Pierpaolo Parravicini, Elisabetta Menatti, Tullio Trotta, Ferdinando Giannattasio, Gabriella Coiro, Fabio Lena, Gianluca Lacerenza, Cristina Mussini, Luisa Tavecchia, Lia Crotti, Gianfranco Parati, Roberto Menè, Maurizio Sanarico, Marco Gori, Francesco Raimondi, Alessandra Stella, Filippo Biscarini, Tiziana Bachetti, Maria Teresa La Rovere, Maurizio Bussotti, Serena Ludovisi, Katia Capitani, Simona Dei, Sabrina Ravaglia, Annarita Giliberti, Giulia Gori, Rosangela Artuso, Elena Andreucci, Angelica Pagliazzi, Erika Fiorentini, Antonio Perrella, Francesco Bianchi, Paola Bergomi, Emanuele Catena, Riccardo Colombo, Sauro Luchi, Giovanna Morelli, Paola Petrocelli, Sarah Iacopini, Sara Modica, Silvia Baroni, Giulia Micheli, Marco Falcone, Donato Urso, Giusy Tiseo, Tommaso Matucci, Davide Grassi, Claudio Ferri, Franco Marinangeli, Francesco Brancati, Antonella Vincenti, Valentina Borgo, Stefania Lombardi, Mirco Lenzi, Massimo Antonio Di Pietro, Francesca Vichi, Benedetta Romanin, Letizia Attala, Cecilia Costa, Andrea Gabbuti, Alessio Bellucci, Marta Colaneri, Patrizia Casprini, Cristoforo Pomara, Massimiliano Esposito, Roberto Leoncini, Michele Cirianni, Lucrezia Galasso, Marco Antonio Bellini, Chiara Gabbi, Nicola Picchiotti, Simone Furini, Chiara Fallerini, Elena Bargagli, Alessandra Renieri

**Affiliations:** 1 Respiratory Disease Unit, Department of Medical Sciences, University Hospital of Siena (Azienda Ospedaliera Universitaria Senese, AOUS), Policlinico Le Scotte, Viale Bracci, 2, 53100 Siena, Italy; 2 Medical Genetics Unit, University of Siena, Policlinico Le Scotte, Viale Bracci, 2, 53100 Siena, Italy; 3 Med Biotech Hub and Competence Center, Department of Medical Biotechnologies, University of Siena, 53100 Siena, Italy; 4 Genetica Medica, Azienda Ospedaliero-Universitaria Senese, 53100 Siena, Italy

**Keywords:** COVID-19, Pulmonary fibrosis, *RTEL1*, Long COVID

## Abstract

**Background:**

Severe Acute Respiratory Syndrome Coronavirus 2 (SARS-CoV-2) is a novel coronavirus that caused an ongoing pandemic of a pathology termed Coronavirus Disease 19 (COVID-19). Several studies reported that both COVID-19 and *RTEL1* variants are associated with shorter telomere length, but a direct association between the two is not generally acknowledged. Here we demonstrate that up to 8.6% of severe COVID-19 patients bear *RTEL1* ultra-rare variants, and show how this subgroup can be recognized.

**Methods:**

A cohort of 2246 SARS-CoV-2-positive subjects, collected within the GEN-COVID Multicenter study, was used in this work. Whole exome sequencing analysis was performed using the NovaSeq6000 System, and machine learning methods were used for candidate gene selection of severity. A nested study, comparing severely affected patients bearing or not variants in the selected gene, was used for the characterisation of specific clinical features connected to variants in both acute and post-acute phases.

**Results:**

Our GEN-COVID cohort revealed a total of 151 patients carrying at least one *RTEL1* ultra-rare variant, which was selected as a specific acute severity feature. From a clinical point of view, these patients showed higher liver function indices, as well as increased CRP and inflammatory markers, such as IL-6. Moreover, compared to control subjects, they present autoimmune disorders more frequently. Finally, their decreased diffusion lung capacity for carbon monoxide after six months of COVID-19 suggests that *RTEL1* variants can contribute to the development of SARS-CoV-2-elicited lung fibrosis.

**Conclusion:**

*RTEL1* ultra-rare variants can be considered as a predictive marker of COVID-19 severity, as well as a marker of pathological evolution in pulmonary fibrosis in the post-COVID phase. This notion can be used for a rapid screening in hospitalized infected people, for vaccine prioritization, and appropriate follow-up assessment for subjects at risk.

*Trial Registration* NCT04549831 (www.clinicaltrial.org)

**Supplementary Information:**

The online version contains supplementary material available at 10.1186/s12931-023-02458-7.

## Background

Severe Acute Respiratory Syndrome Coronavirus 2 (SARS-CoV-2) is a new coronavirus that became pandemic in 2019. The disease caused by this virus was named Coronavirus Disease 2019 (COVID-19) [1]. The course of SARS-CoV-2 infection is unpredictable, with symptoms ranging from absent to severe, sometimes even with a lethal outcome [2].

In addition to demographic risk factors, such as old age and/or male sex, the neutrophil to lymphocyte ratio (NLR) has been shown to have the greatest predictive value for poor outcomes in patients with COVID-19. Genetic markers of severity and susceptibility to infection were also considered [3, 4]. In particular, telomere shortening is associated with a higher risk of developing severe COVID-19 [5]. Different studies reported that COVID-19 associates with shorter telomere length, revealing that severe COVID-19 survivors have shorter telomeres compared with patients recovered from milder COVID-19 [5, 6]. The critical shortness of telomeres results from permanent DNA damage, with the induction of cell senescence and apoptosis [7]. Several human pathologies are characterized by telomere shortening. Fibrosis in the lung, liver, or kidney is often associated with dysfunction in telomere-binding proteins and generally with pathogenic variants in genes relevant to the homeostasis of telomeres, such as *RTEL1* [8].

Pathogenic variants in *RTEL1* gene, encoding for a helicase that regulates telomere elongation, have been identified in rare interstitial pneumoniae, called Idiopathic Pulmonary Fibrosis (IPF) [9]. Moreover, *RTEL1*-mutated pulmonary fibrosis families display a precocious onset of pulmonary disease, concomitant liver pathologies, and in some cases early reversible neutropenia [10]. Some of these patients also present autoimmune conditions, suggesting that, in heterozygous carriers of *RTEL1* aberrations, fibrosis results from the combination of such monogenic defects with environmental factors and autoimmune diseases [11, 12]. Cellular and molecular pathways, including TGF-beta and IL-6 over-production [13, 14], are shared between IPF and COVID-19. From a genetic point of view, GWAS studies identified some tens of quantitative *loci* involved in COVID-19 severity/susceptibility [3]. Common, low-frequency, rare, and ultra-rare coding variants were also found to contribute to COVID-19 severity [15]. Limited data are now available regarding telomere length and COVID-19 progression [16, 17]; *RTEL1* variants, however, have never been investigated as a possible mechanistic connection between the two. This study aims to describe the clinical characteristics of an Italian cohort of COVID-19 patients, either bearing ultra-rare variants of *RTEL1* or not.

## Materials and methods

### Study design and populations

A cohort of 2246 SARS-CoV-2-positive subjects, collected within the GEN-COVID Multicenter study (https://sites.google.com/dbm.unisi.it/gen-covid), was used in this work. The application of the post-Mendelian model allowed us to extract the genetic features contributing to the COVID-19 phenotype [15]. Patients were classified using a modified version of the World Health Organization COVID-19 outcome scale [18]. The following six categories of severity were identified: (1) death; (2) hospitalized, receiving invasive mechanical ventilation; (3) hospitalized, receiving continuous positive airway pressure (CPAP) or bilevel positive airway pressure (BiPAP) ventilation; (4) hospitalized, receiving low-flow supplemental oxygen; (5) hospitalized, not receiving supplemental oxygen; and (6) not hospitalized. In order to obtain a clinical classification as independent as possible from age and sex, that allowed us to define a cohort in which the genetic features were more relevant to determine the severe/mild phenotype, we performed an adjustment starting from the clinical categories. We applied two ordered logistic regression, separately for males and females cohort, and cases who received a treatment higher than expected by age were classified as severe, while patients who received a treatment less severe than expected by age were considered not severe; subjects matching the expected treatment outcomes according to age were excluded from the model [20]. A subset of 512 COVID-19 patients, for which all clinical and laboratory parameters were available, was selected**.** This subset of patients is stratified based on *RTEL1* genotype: a case group of 151 mutated patients (126 hospitalized patients and 25 not-hospitalized patients) is defined, composed of 92 males and 59 females from various regions of Italy; 361 non-mutated patients became our control group, subdivided among 222 males and 139 females and monitored at the COVID-19 wards of the Siena University Hospital from March 1st, 2020 to July 1st, 2021. SARS-CoV-2 positivity was confirmed by a nasopharyngeal antigenic swab, performed upon admission. All data were collected prospectively at the time of hospitalization and gathered in an electronic database in anonymous form. For each patient clinical, radiological, immunological, laboratory, and survival information has been collected. Functional data, including the percentages of forced vital capacity (FVC) and the diffusing capacity of the lung for carbon monoxide (DLCO), were also collected at 6 (± 1) months of follow-up, monitored at Siena University Hospital. This longitudinal study was conducted in a sub-subset of the 512 cohort, namely, *RTEL1* mutated patients attending Siena hospital (12) and 18 non-mutated patients matched for age and sex. The GEN-COVID Multicenter study was performed in accordance with all relevant international, European, Italian, and institutional guidelines, and approved in advance by the University Hospital (Azienda Ospedaliero-Universitaria Senese) Ethical Review Board, Siena, Italy (Prot n. 16917, dated March 16th, 2020).

### Whole exome sequencing (WES) analysis

WES was performed using the NovaSeq6000 System (Illumina, San Diego, CA, USA) with at least 97% coverage at 20×, as previously described [19]. Data were represented in a binary mode on a gene-by-gene basis [15, 19, 20].

### Statistical analysis

The LASSO logistic regression machine learning approach used in the post-Mendelian model allow us to extract relevant genetic features associated with COVID-19 clinical outcome, as already described [15, 20].

In this study, we consider only ultra-rare (Minor Allele Frequency < 0.001) autosomal dominant gene variants (presence of at least one variant) as Boolean features.

Clinical data were stored in Microsoft Excel. Results were expressed as means plus/minus a standard deviation (M ± SD), or medians and quartiles (25th and 75th percentiles) for continuous variables as necessary. The Shapiro–Wilk test was applied to evaluate the normal distribution of data. Chi-square tests or Fisher exact tests were used for categorical variables as appropriate. Comparisons between control and patient groups were conducted by Student’s *t*-test or Mann–Whitney *U* test, while for multiple comparisons a one-way ANOVA or non-parametric tests (Kruskal–Wallis test and Dunn test) were performed. Statistical analysis and graphic representation of the data were performed using dedicated software, namely GraphPad Prism 9.4.2 (Graphpad Holdings, LLC, San Diego, CA, USA) and Jamovi (version 1.8.1) [Computer Software] (Retrieved from https://www.jamovi.orgc). For all tests, *p*-values of less than 0.05 were considered statistically significant.

## Results

### *RTEL1* ultra-rare variants associate with severity in COVID-19

The LASSO logistic regression extracted *RTEL1* ultra-rare variants as one of the most important features associated with severity [15]. Exome analysis of 2246 SARS-CoV-2 infected subjects of different severity, belonging to GEN-COVID cohort, stratified by sex and adjusted by age, shows an association between *RTEL1* ultra-rare variants and severity with an OR = 1.63 (95% CI 1.04 to 2.58; p-value = 0.03), (Table 1). In the total cohort, 151 patients carried one *RTEL1* ultra-rare variant, 126 being hospitalized, and 25 being not. The specific variants are illustrated in Additional file 1: Table S1a, b.

**Table 1:** Chi Square test in the GEN-COVID cohort

Phenotype	Ultra-rare variants (%)	Wild type (%)	Total
Severe	59 (66.3)	931 (54.7)	990
Not severe	30 (33.7)	772 (45.3)	882
Total	89	1703	1792

OR = 1.63, (95% CI 1.04–2.58), p-value = 0.03

### *RTEL1* mutated patients are younger and require more respiratory support and duration of hospitalization

Demographic, clinical and survival data are reported in Table 2. No differences in terms of gender distribution, in the frequencies of bilateral pneumonia on chest X-ray, and in survival rate are identified relating to *RTEL1* genotype. *RTEL1* mutated patients, on the other hand, result to be younger than other patients, with fewer duration of hospitalization. Similarly, the percentage of patients that required respiratory support with CPAP during hospitalization is significantly higher among wildtype (WT) patients than the cohort of patients with *RTEL1* ultra-rare variants, because the latter underwent more often intubation.

**Table 2:** Demographic, Clinical and survival data

	RTEL1 mutation	RTEL1 WT	p values
Gender (M/F) (% of male)	92/59 (61)	222/139 (61)	ns
Age (M ± S.D.)	59.11 ± 16.32	65.5 ± 14.22	< 0.0001
Days of Hospitalization (M ± S.D.)	22.15 ± 16.08	27.28 ± 34.48	0.048
Bilateral pneumoniae (yes/no) (%yes)	51 (34)	137 (38)	ns
Oxygen Administration (yes/no) (%yes)	104 (69)	332 (92)	0.0023
Type of oxygen Therapy			
Nasal cannula (%)	72 (48)	166(46)	0.0117
CPAP/High Flows (%)	46 (31)	148 (41)	
Intubation (%)	33 (21)	46 (13)	
Survival (Death) (%death)	12 (8%)	22 (6%)	ns
Number of comorbidities			ns
No comorbidities	59	46	
1	18	28	
2	12	13	
3	10	9	
> 4	1	4	

### *RTEL1*-mutated patients have more autoimmune diseases as comorbidity

The number and type of comorbidities in *RTEL1*-mutated versus WT individuals are reported in Table 2. The number of comorbidities affecting each patient is similar for the two groups. However, *RTEL1*-mutated patients are more affected by autoimmune diseases (12% in mutated patients and 6% in WT patients) and hypertension (40% in mutated patients and 29% in WT patients) compared to other patients. Diabetes, lung disease, cancer, dyslipidemia, and hypothyroidism show similar incidences in the two cohorts.

### Impaired liver function and NLR

Concerning laboratory findings, total bilirubin, Alanine aminotransferase (ALT), and IL-6 levels in the blood are significantly higher in the population with *RTEL1* variants. The concentrations of Aspartate aminotransferase (AST) showed a similar trend, although without reaching statistical significance (Table 3).

**Table 3:** Laboratories parameters of analyzed cohort

	RTEL1 mutation	RTEL1 WT	p values
Total bilirubin (mg/dl)	20.91 ± 42.17	15.62 ± 3.076	< 0.0001
AST (U/L)	53.99 ± 61.09	48.15 ± 82.9	0.0555
ALT (U/L)	63.11 ± 55.61	54.61 ± 74.70	0.0084
Creatinine (mg/dl)	2.2 ± 8.8	1.26 ± 3.34	0.0512
D-Dimer (ng/dl)	1888 ± 2774	2722 ± 7040	0.0534
Fibrinogen (mg/dl)	485.3 ± 203.7	597.6 ± 160.9	0.0007
IL-6 (pg/ml)	17.35 ± 33.29	6.46 ± 16.69	0.0159
Platelets (10^3^/mm^3^)	208.3 ± 82.64	222.6 ± 101.7	ns
CRP (mg/dl)	51.35 ± 131	83.9 ± 276.3	< 0.0001
LDH (U/L)	428.4 ± 329.2	348.1 ± 370.2	ns
Lypase (U/L)	56.37 ± 52.32	65.14 ± 18.5	ns
Pancreatic Amylase (U/L)	46.65 ± 22.16	56.65 ± 83.64	ns
Gamma Glutamin Transferase (U/L)	86.29 ± 103.8	70.69 ± 133.4	ns

With respect to controls, carriers of *RTEL1* ultra-rare variants also showed a significant decrease in creatinine, D-dimer, fibrinogen, and c-reactive protein (CRP), (Table 3).

**Fig. 1:**
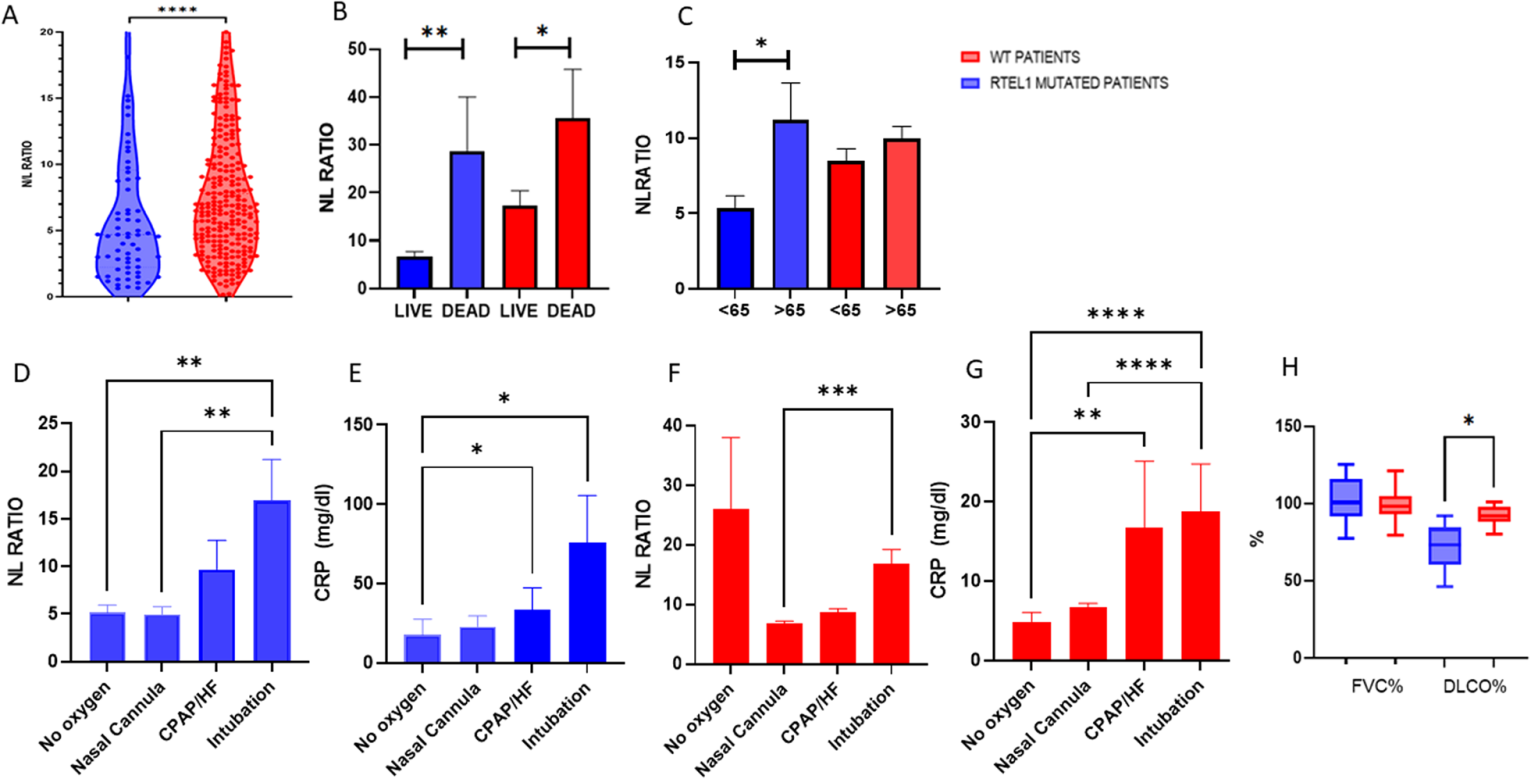
**A** Values of N/L ratio in WT (red) and *RTEL1*-mutant (blue). **B** Values of N/L ratio after stratification for survival rate (dead/live) and **C** for age (< 65 years and > 65 years) in WT (red) and *RTEL1*-mutant (blue). **D** The values of N/L ratio and **E** CRP in *RTEL1*-mutant after the stratification based on ventilatory support. **F** The values of N/L ratio and **G** CRP in WT patients after the stratification based on ventilatory support. **H** The percentages of FVC and DLCO in *RTEL1*-mutant versus WT individuals after 6 months of follow-up. *NL* neutrophil to lymphocytes, *HF* high flows. *p < 0.05 **p < 0.01 ***p < 0.001 ****p < 0.0001

As Fig. 1A shows, NLR is significantly higher in the unmutated population. Particularly after cohort stratification by survival rate, higher NLRsreported in dead patients irrespective of their *RTEL1* genotype (Fig. 1B). Interestingly, grouping by age highlighted that patients older than 65 years and bearing an ultra-rare *RTEL1* variant had higher values of these parameters (Fig. 1C).

NLR and CRP levels were also analyzed based on ventilatory support. Among *RTEL1-*mutated patients, NLR is significantly higher in intubated, compared to those who did not require oxygen or used nasal cannulas; in the same way, CRP is relevantly more concentrated in patients requiring either CPAP ventilation or intubation (Fig. 1D, E).

NLR did not correlate with ventilatory support in the WT population, while CRP showed the same trend reported for *RTEL1*-mutated, namely showing higher values for intubated and CPAP-treated patients (Fig. 1F, G).

### Decreased diffuse lung capacity in *RTEL1*-mutated patients at six months post-SARS-CoV-2 infection

To understand the role of *RTEL1* on fibrotic development, post-COVID-19 pulmonary function tests were performed in a group of twelve patients with ultra-rare *RTEL1* variants. Exams included FVC, DLCO, and laboratory analyses. The cohort was matched for age and sex to 18 patients with WT *RTEL1* variants. Comparison analyses unveiled a relevant DLCO decrease for *RTEL1* mutants (71.8 ± 14.4% versus 92.12 ± 5.6, respectively; *p* = 0.02). No differences are found for other parameters under consideration.

## Discussion

The current COVID-19 pandemic represents a major public health concern, with more than 600 million cases worldwide at the time of writing (https://covid19.who.int/). The number of survivors improved in the last period, due to the development and deployment of vaccinations and other treatments [21]. Follow-up pneumological evaluations on people surviving severe COVID-19 evidenced a 40% chance of developing pulmonary sequelae with the potential for a neat decrease in life quality [22].

The development of pulmonary fibrosis following COVID-19 remains an open challenge for research. Little information is present in the literature, and no clear correlation emerged between the severity of COVID-19 and the development of fibrosis within the first year of post-infection monitoring [23]. Known shared molecular pathways between pulmonary fibrosis and COVID-19 are almost limited to those pertaining aberrant inflammation in association with dysregulated repair mechanisms and fibrogenesis [14]. Regarding genetic alterations, some similarities emerged between pulmonary fibrosis and altered lung functions following COVID-19. *MUC5B* and *SFTPD* are considered interesting *loci* associated with COVID-19 severity, and both genes are strongly correlated with pulmonary fibrosis onset [24]. Shorter telomeres are linked to worse COVID-19 symptoms, among which appears a delayed resolution of radiographic lung abnormalities [6, 25]. Telomere shortening is consistently observed in older adults, and therefore it is considered a reliable marker of aging associated with an increased risk of developing cardiovascular diseases and other disorders [26], including pulmonary fibrosis [27]. In COVID-19, shorter telomeres in peripheral blood cells are associated with less favorable prognosis [5], while this aspect does not seem to be influenced by age in post-COVID-19 analyses, possibly indicating that SARS-CoV-2 infection reduces telomere length directly [6].

In this paper, we demonstrated a relationship between *RTEL1* genotype and COVID-19 phenotype, both in the acute and post-acute phase of the disease. Genetic alterations on *RTEL1* may account for up to 8.6% of hospitalized patients. Recent but sound evidence identifies the gene as a major driver of interstitial lung disease (ILD) and heterozygous variants have been reported in about 5–9% of familial ILD [12, 28]. Our interpretation of these data is that SARS-CoV-2 infection triggers the underneath genetic susceptibility due to *RTEL1* variants, leading to both a need for higher respiratory support in the acute phase, as well as to a chronic fibrotic process, which may eventually result in open ILD depending on specific (often private) variant (see Additional file 1: Table S1).

Our analyses found that *RTEL1*-mutated patients are younger, although with comparatively prolonged hospitalization and more frequent need for invasive ventilation. We believe this all makes *RTEL1* a valid prognostic marker for COVID-19. Moreover, the mutated cohort more often presented impaired liver function and undesirable NLR in peripheral blood. Borie et al*.*, described liver involvement for ILD subjects with *RTEL1* alterations. Interestingly, these patients are seemingly less prone to develop pathological extrapulmonary phenotypes [29].

Both neutropenia and lymphocytosis can result in an NLR decrease. Previous studies do not generally mention neutropenia and lymphocytosis as a manifestation of COVID-19 infection. To the best of our knowledge, the phenomenon is only described in a few case reports [30, 31], or in patients with a concomitant hematological malignancy or solid tumor [32–34]. Kannengiesser et al*.* describe an early reversible neutropenia after cyclophosphamide treatment in pulmonary fibrosis patients with *RTEL1* variants [10]. Among hematological abnormalities, the incidence of lymphopenia ranged from 40 to 80%, with increased NLR [35] associated with higher mortality rates, especially for severe cases of COVID-19 [36, 37]. We hypothesize that NLR alterations stem from the invasiveness of ventilation procedures. Exclusively for *RTEL1* mutated, NLR trends reflected ventilation treatments better than CRP, which is currently the most used prognostic biomarker for COVID-19 [38–40].

Our *RTEL1*-mutated patients showed a higher prevalence of autoimmune COVID-19 comorbidities. This is in line with results from another cohort of individuals concomitantly presenting interstitial pneumoniae, autoimmune diseases, and *RTEL1* variants in heterozygosity [41]: similarly to COVID-19 population, such patients also showed clinical manifestation related to a telomere syndrome, such as hematological abnormalities (*i.e.*, neutrophil and lymphocyte alterations) and liver pathologies, along with an earlier manifestation of the pulmonary disease [42, 43].

Finally, the decreased DLCO after six months of COVID-19 suggests that *RTEL1* variants can contribute to the development of lung fibrosis following COVID-19. However, this data needs to be confirming in a largest cohort also considering HRCT and other clinical and functional parameters. It is well demonstrated that in the majority of patients, DLCO and respiratory symptoms tend to normalize or improve one year after hospitalization [44]. There are, however, about 33% cases in which respiratory dysfunction persists, requiring prolonged follow-up. *RTEL1* variants are reportedly the first genetic risk factor for the prediction of lung impairment after COVID-19. A wider cohort is needed for an accurate early identification of these patients.

## Conclusion

In conclusion, our findings establish shared clinical risk factors between COVID-19 and pulmonary fibrosis. *RTEL1* ultra-rare variants can be considered as a predictive marker of COVID-19 severity, as well as a marker of pathological evolution for pulmonary fibrosis in the post-COVID phase. This notion can be exploited for rapid screening in hospitalized infected people, for vaccine prioritization, and for appropriate follow-up assessment in subjects at risk.

## Supplementary Information

**Additional file 1. Table S1a**. *RTEL1* ultra-rare variants in hospitalized COVID-19 patients. **Table S1b**. *RTEL1* ultra-rare variants in not-hospitalized COVID-19 patients.

## Data Availability

The datasets used and/or analysed during the current study are available from the corresponding author on reasonable request.
