# Supplementary material for: Ultra-rare RTEL1 gene variants associate with acute severity of COVID-19 and evolution to pulmonary fibrosis as a specific long COVID disorder
Source: Respir Res. 2023 Jun 16;24:158. doi: 10.1186/s12931-023-02458-7 (PMC10276396; doi:10.1186/s12931-023-02458-7)
Supplement: Supplementary file 1 — Additional file 1. Table S1a. RTEL1 ultra-rare variants in hospitalized COVID-19 patients. Table S1b. RTEL1 ultra-rare variants in not-hospitalized COVID-19 patients. [file 12931_2023_2458_MOESM1_ESM.docx]

| **Nucleotide change** | **Amino acid change** | **dbSNP** | **CADD** | **ExAC_NFE** | **tot. n. patients** | **Sex (n.)** | **Age range** | **Category** † |
| --- | --- | --- | --- | --- | --- | --- | --- | --- |
| c.77C>T | p.T26I | rs773115541 | 18.8 | 0,00003 | 1 | M | 51 | 5 |
| c.3676G>A | p.A1226T | rs371373011 | 11.4 | 0,00005 | 1 | M | 74 | 1 |
| c.3725G>A | p.C1242Y | rs765990015 | 0.11 | 0,00003 | 1 | F | 77 | 1 |
| c.334G>A | p.A112T | rs151214675 | 28.3 | 0,0003 | 2 | 1 M | 34 | 5 |
|  |  |  |  |  |  | F | 70 | 4 |
| c.463A>G | p.S155G | rs762388949 | 18.6 | 0.000009 | 2 | F (2) | 57-66 | 4-3 |
| c.509G>A | p.R170H | rs750794827 | 24.2 | 0,00002 | 1 | M | 72 | 4 |
| c.637C>T | p.R213W | rs560281693 | 34 | 0.000009 | 1 | M | 59 | 3 |
| c.301C>T | p.R101C | rs771452777 | 24.2 | 0,00006 | 1 | M | 82 | 3 |
| c.349G>A | p.G117S | rs145845927 | 27 | 0,0008 | 2 | F | 47 | 3 |
|  |  |  |  |  |  | M | 78 | 3 |
| c.395C>T | p.A132V | n.a. | 26.1 | n.a. | 1 | F | 78 | 5 |
| c.520C>G | p.Q174E | rs150285674 | 24.3 | 0,0006 | 6 | F (4) | 47-75 | 5-1 |
|  |  |  |  |  |  | M (2) | 47-56 | 5-4 |
| c.632C>T | p.T211M | rs77086616 | 16.3 | 0,0004 | 3 | F (2) | 46-58 | 5-4 |
|  |  |  |  |  |  | M | 39 | 4 |
| c.662C>G | p.T221S | rs763825991 | 3.3 | 0,00005 | 1 | M | 68 | 3 |
| c.675G>C | p.K225N | rs755129499 | 19.3 | n.a. | 1 | M | 33 | 4 |
| c.850G>A | p.D284N | rs778782744 | 21.1 | 0.000008 | 1 | M | 44 | 1 |
| c.934G>A | p.E312K | n.a. | 22.9 | n.a | 1 | M | 76 | 2 |
| c.1006T>A | p.F336I | rs747497376 | 28.5 | 0,00002 | 2 | M | 78 | 1 |
|  |  |  |  |  |  | F | 92 | 1 |
| c.1286T>C | p.M429T | rs148080505 | 13.5 | 0,0003 | 1 | F | 72 | 3 |
| c.1592G>A | p.R531Q | rs141423196 | 13.6 | 0,0002 | 2 | F | 87 | 4 |
|  |  |  |  |  |  | M | 74 | 3 |
| c.1677_1679del | p.F561del | rs771771098 | n.a. | 0,00002 | 2 | M (2) | 56-75 | 4 |
| c.1604C>T | p.A535V | rs745535053 | 3.1 | n.a. | 1 | M | 65 | 3 |
| c.1637G>A | p.R546H | rs369014080 | 13.9 | 0,0001 | 5 | M (4) | 47-70 | 1-3 |
|  |  |  |  |  |  | F (1) | 46 | 5 |
| c.1769A>G | p.E590G | rs756711633 | 12.3 | n.a. | 1 | M | 44 | 4 |
| c.1801C>T | p.P601S | rs138188555 | 0.03 | 0,0006 | 2 | M (2) | 67-76 | 3-1 |
| c.1813C>T | p.R605W | rs376930232 | 24.1 | 0,00003 | 1 | M | 56 | 2 |
| c.1876G>A | p.G626S | rs908799108 | 2.1 | n.a. | 1 | M | 61 | 4 |
| c.1931C>T | p.P644L | rs139083375 | 3.8 | 0,0006 | 3 | F (3) | 51-87 | 5 |
| c.1943C>T | p.P648L | rs144002969 | 0.9 | 0,0002 | 2 | F (2) | 63-67 | 3 |
| c.1955G>A | p.R652K | rs200565373 | 4.8 | 0,00003 | 2 | F (2) | 69-80 | 1-3 |
| c.2009C>T | p.T670M | rs141717966 | 4.7 | 0,0003 | 1 | M | 56 | 3 |
| c.2141C>T | p.P714L | rs140986557 | 13.9 | n.a. | 1 | M | 69 | 3 |
| c.2159C>T | p.P720L | rs142969505 | 24.8 | 0,00008 | 3 | F (2) | 71-73 | 3-4 |
|  |  |  |  |  |  | M | 69 | 4 |
| c.1993G>C | p.V665L | rs200505378 | 6.3 | n.a. | 1 | M | 32 | 3 |
| c.G2132C | p.C711S | n.a. | 0.001 | n.a. | 1 | M | 65 | 5 |
| c.2149G>A | p.A717T | rs750353477 | 15.4 | 0,00002 | 1 | M | 70 | 5 |
| c.2154G>C | p.E718D | rs61736614 | 22.1 | 0,0003 | 8 | M (6) | 29-78 | 4-5 |
|  |  |  |  |  |  | F (2) | 7m.- 53 | 4 |
| c.2200C>T | p.R734W | rs398123018 | 33 | 0,00006 | 1 | M | 80 | 4 |
| c.2208C>A | p.H736Q | rs373996455 | 8.6 | 0,0001 | 1 | F | 77 | 4 |
| c.2246C>T | p.T749I | rs199834369 | 18.7 | n.a. | 2 | M | 25 | 4 |
|  |  |  |  |  |  | F | 50 | 5 |
| c.2284C>T | p.P762S | rs772031890 | 4.7 | n.a | 1 | M | 74 | 3 |
| c.2285C>T | p.P762L | n.a. | 22.9 | n.a. | 1 | M | 49 | 4 |
| c.2296C>T | p.R766W | rs139221232 | 18.6 | 0,0003 | 1 | F | 85 | 1 |
| c.2300C>T | p.A767V | rs370171864 | 9.7 | 0,00009 | 2 | M (2) | 64-72 | 5-2 |
| c.2306C>T | p.P769L | rs143967591 | 10.9 | 0,0009 | 13 | M (11) | 46-93 | 5-1 |
|  |  |  |  |  |  | F (2) | 58-87 | 4-1 |
| c.2197G>C | p.V733L | rs942820151 | 23 | n.a. | 1 | F | 66 | 4 |
| c.2226G>C | p.E742D | rs745327960 | 1.4 | n.a. | 1 | M | 65 | 3 |
| c.2234G>C | p.C745S | rs375503989 | 23.8 | 0,00008 | 1 | M | 19 | 5 |
| c.2387A>G | p.Q796R | rs116053476 | 0.2 | 0,0002 | 3 | F | 55 | 5 |
|  |  |  |  |  |  | M (2) | 57-59 | 4-5 |
| c.2353G>A | p.V785M | rs145335410 | 0.02 | 0,0002 | 1 | F | 58 | 4 |
| c.2368G>A | p.A790T | rs142568980 | 0.13 | n.a. | 1 | M | 69 | 3 |
| c.2533C>T | :p.R845C | rs762640318 | 26.7 | n.a. | 1 | M | 76 | 5 |
| c.2485G>A | p.A829T | n.a. | 8.6 | n.a. | 1 | M | 55 | 3 |
| c.2506G>A | p.A836T | rs115303435 | 12.8 | 0,00006 | 2 | F | 48 | 4 |
|  |  |  |  |  |  | M | 69 | 4 |
| c.2515G>A | p.A839T | rs773397014 | 1.62 | 0,00005 | 1 | M | 41 | 3 |
| c.2534G>A | p.R845H | rs137914057 | 11.9 | 0,0004 | 2 | M | 85 | 3 |
|  |  |  |  |  |  | F | 72 | 3 |
| c.2720A>G | p.Q907R | n.a. | 21.8 | n.a. | 1 | M | 89 | 3 |
| c.2723C>T | p.T908M | rs372852392 | 12.9 | 0,00003 | 1 | M | 67 | 4 |
| c.2711G>A | p.R904 | rs771768208 | 19 | 0,00002 | 1 | M | 55 | 3 |
| c.2786G>A | p.R929K | rs763777407 | 4.2 | 0,00002 | 2 | M | 53 | 2 |
|  |  |  |  |  |  | F | 66 | 4 |
| c.2787G>T | p.R929S | n.a. | 0.001 | n.a. | 1 | F | 66 | 4 |
| c.2794G>A | p.V932M | rs149145821 | 0.05 | 0,0001 | 1 | F | 75 | 1 |
| c.2839C>T | p.R947W | rs773894104 | 13.6 | 0,00003 | 1 | M | 61 | 2 |
| c.2852C>T | p.T951I | rs201992738 | 9.4 | 0,00003 | 2 | M (2) | 62-63 | 2 |
| c.2954C>T | p.P985L | rs1020833078 | 23.4 | n.a. | 1 | F | 7m | 4 |
| c.2920G>A | p.G974S | rs545606632 | 7.8 | 0,00006 | 1 | M | 58 | 3 |
| c.2921G>C | p.G974A | rs138500086 | 11.4 | 0,0001 | 1 | M | 66 | 3 |
| c.2923G>A | p.E975K | rs771457769 | 3.4 | 0,0002 | 8 | M (6) | 50-82 | 4-2 |
|  |  |  |  |  |  | F (2) | 67-75 | 2 |
| c.2836T>C | p.S946P | rs143248833 | 0.12 | 0,0002 | 2 | M (2) | 19-59 | 2-5 |
| c.2878T>A | p.S960T | rs745305797 | 0.005 | 0,00002 | 1 | F | 58 | 4 |

**Table S1a.** *RTEL1* ultra-rare variants in hospitalized COVID-19 patients

†Clinical category: 1, death; 2, hospitalized, receiving invasive mechanical ventilation; 3, hospitalized, receiving continuous positive airway pressure (CPAP) or bilevel positive airway pressure (BiPAP) ventilation; 4, hospitalized, receiving low-flow supplemental oxygen; 5, hospitalized, not receiving supplemental oxygen; 6, not hospitalized; m. = months; CADD, Combined Annotation Dependent Depletion; ExAC_NFE, Non-Finnish European minor allele frequency; n.a., not available.

| **Nucleotide change** | **Amino acid change** | **dbSNP** | **CADD** | **ExAC_NFE** | **tot. n. patients** | **Sex (n.)** | **Age range** | **Category** † |
| --- | --- | --- | --- | --- | --- | --- | --- | --- |
| c.302G>A | p.R101H | rs113684274 | 17.6 | 0,0002 | 1 | M | 45 | 6 |
| c.3734A>G | p.Q1245R | n.a. | 0.001 | n.a. | 1 | F | 55 | 6 |
| c.349G>A | p.G117S | rs145845927 | 27 | 0,0008 | 1 | M | 37 | 6 |
| c.520C>G | p.Q174E | rs150285674 | 24.3 | 0,0006 | 1 | F | 64 | 6 |
| c.632C>T | p.T211M | rs77086616 | 16.3 | 0,0004 | 2 | M (2) | 30-57 | 6 |
| c.1193C>T | p.A398V | rs535749230 | 33 | 0,00001541 | 1 | M | 29 | 6 |
| c.G1402G>A | p.V468M | n.a. | 28.4 | n.a. | 1 | F | 46 | 6 |
| c.1637G>A | p.R546H | rs369014080 | 13.9 | 0,0001 | 2 | F (2) | 31 | 6 |
| c.1876G>A | p.G626S | rs908799108 | 2.1 | n.a. | 2 | M (1) | 49 | 6 |
|  |  |  |  |  |  | F (1) | 45 | 6 |
| c.1943C>T | p.P648L | rs144002969 | 0.9 | 0,0002 | 1 | F | 48 | 6 |
| c.2155G>A | p.D719N | rs116247954 | 25.7 | n.a. | 1 | F | 31 | 6 |
| c.2296C>T | p.R766W | rs139221232 | 18.6 | 0,0003 | 1 | F | 38 | 6 |
| c.2306C>T | p.P769L | rs143967591 | 10.9 | 0,0009 | 3 | M | 32 | 6 |
|  |  |  |  |  |  | F (2) | 19-66 | 6 |
| c.2311C>G | p.L771V | n.a. | 3.6 | n.a. | 1 | F | 59 | 6 |
| c.2297G>T | p.R766L | n.a. | 13.6 | n.a | 1 | F | 30 | 6 |
| c.2551G>A | p.A851T | rs780364760 | 8,8 | 0,00006 | 1 | M | 41 | 6 |
| c.2776C>T | p.Q926X | n.a. | 35 | n.a. | 1 | F | 53 | 6 |
| c.2920G>A | p.G974S | rs545606632 | 7.8 | 0,00006 | 2 | F (2) | 62 | 6 |
| c.2836T>C | p.S946P | rs143248833 | 0.12 | 0,0002 | 1 | M | 60 | 6 |

**Table S1b.** *RTEL1* ultra-rare variants in not-hospitalized COVID-19 patients

†Clinical category: 1, death; 2, hospitalized, receiving invasive mechanical ventilation; 3, hospitalized, receiving continuous positive airway pressure (CPAP) or bilevel positive airway pressure (BiPAP) ventilation; 4, hospitalized, receiving low-flow supplemental oxygen; 5, hospitalized, not receiving supplemental oxygen; 6, not hospitalized; m. = months; CADD, Combined Annotation Dependent Depletion; ExAC_NFE, Non-Finnish European minor allele frequency; n.a., not available.
